# Localized propranolol delivery from a copper-loaded hydrogel for enhancing infected burn wound healing via adrenergic β-receptor blockade

**DOI:** 10.1016/j.mtbio.2024.101417

**Published:** 2024-12-20

**Authors:** Wenzhe Sun, Hongwei Lu, Pengqin Zhang, Lian Zeng, Bing Ye, Yi Xu, Jianan Chen, Peiran Xue, Jialin Yu, Kaifang Chen, Bin Wu, Xiao Lv, Xiaodong Guo, Yanzhen Qu

**Affiliations:** Department of Orthopaedics, Union Hospital, Tongji Medical College, Huazhong University of Science and Technology, Wuhan, Hubei Province, China

**Keywords:** Burn wound healing, Hydrogel, Angiogenesis, Propranolol, Sympathetic nerves, Infection

## Abstract

Severe burn injuries immediately trigger a sustained systemic and local stress response. During this process, the sympathetic nervous system releases large amounts of catecholamines, which bind to β-adrenergic receptors (β-AR) on cell membranes, negatively affecting skin regeneration. Additionally, recurrent bacterial infections make burn wounds difficult to treat, posing significant and ongoing challenges to burn care. To address these challenges, we pioneered the study of locally delivered propranolol for burn wound treatment, revealing its ability to antagonize norepinephrine (NE) and regulate the sympathetic nervous system. In this study, a Cu^2^⁺-loaded anti-sympathetic hydrogel (copper ion cross-linked propranolol@gelatin/alginate, PNL@GA-Cu) was developed to remodel the challenging neuromodulatory microenvironment and accelerate the repair of the infected burn wound. The hydrogel system releases Cu^2^⁺ and propranolol simultaneously during degradation, synergistically acting on local wound tissue. Cu^2^⁺ exhibits dual effects of antibacterial activity and promoting angiogenesis, effectively killing *Staphylococcus aureus* and *Escherichia coli* while enhancing the expression of angiogenesis-related genes (CD31, VEGF). Meanwhile, propranolol can counteract the inhibitory effects of NE simulated chronic stress microenvironment on angiogenesis and mitigate sympathetic nerve innervation during the early stages of wound healing. Finally, the PNL@GA-Cu hydrogel significantly promoted the repair of third-degree full-thickness burns in SD rats. Approaches targeting the neural microenvironment for burn wound treatment has not been previously addressed in the literature. The anti-sympathetic PNL@GA-Cu hydrogel offers a promising strategy for treating infected burn wounds. Remodeling the neuromodulatory microenvironment could be an emerging strategy in tissue engineering.

## Highlights

• Copper ion cross-linked hydrogels exhibit enhanced stability, porosity and mechanical strength.
• Propranolol regulates sympathetic nerve activity by affecting tyrosine hydroxylase expression and migration of PC12 cells.
• Propranolol released from PNL@GA-Cu hydrogel can reverse the inhibitory effect of norepinephrine on angiogenesis.
• Cu^2+^ released from PNL@GA-Cu hydrogels exhibit dual effects of promoting angiogenesis and antibacterial effects.
• PNL@GA-Cu hydrogel significantly promoted the repair of third-degree full-thickness burns in SD rats.

## Graphical abstract

Schematic diagram of the process of copper-loaded anti-sympathetic hydrogel (PNL@GA-Cu) promoting the healing of infected burn wounds.

**Figure:**
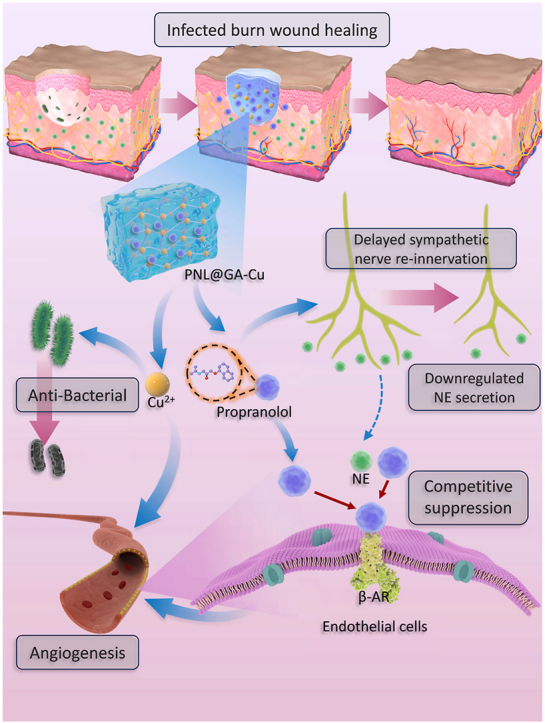

## 1 Introduction

Burn injury, typically caused by exposure to flames, hot liquids, hot gases, chemicals, or electricity, is the fourth most common traumatic injury worldwide, affecting 11 million people annually, with 180,000 cases being fatal [1,2]. Skin grafting remains the gold standard for treating severe burn wounds in clinics to lower mortality rates and shorten hospital stays [3]. However, challenges such as donor skin scarcity, graft fragility, rough texture, and increased scar contractures restrict the practical application of autologous skin grafting [4]. Furthermore, severe burns are difficult to manage due to recurrent bacterial infections and metabolic alterations, leading to hypertrophic scarring, ulceration, and potentially necessitating amputation, posing a significant ongoing challenge in burn care [5]. These factors make the treatment process for burn patients complex and prolonged. Compared to other types of wounds such as diabetic ulcers, burns exhibit a unique hypermetabolic response which is closely associated with increased catabolism, organ failure, heightened infection risks, and even elevated mortality rates.

Unlike sepsis or trauma, severe burns elicit an immediate systemic and local stress response that does not quickly subside and can persist for several years. Recent studies have shown that soft tissue damage triggers the release of large amounts of catecholamine hormones from sympathetic nerves. These hormones bind to β-adrenergic receptors (β-ARs) on cell membranes, adversely affecting skin regeneration [[6], [7], [8]]. Nearly fifty years ago, Wilmore et al. ingeniously demonstrated that catecholamines mediate the hypermetabolic response following thermal injury [9]. β-blockers, particularly propranolol (PNL), specifically inhibit the activation of β-AR in both acute and chronic skin injuries. This leads to reduced inflammation duration, accelerated keratinocyte migration, enhanced wound contraction and angiogenesis, and inhibition of bacterial virulence, thereby promoting soft tissue wound healing [8]. Moreover, β-blockers have been shown to expedite wound healing in patients with severe thermal injuries by mitigating the hypermetabolic response [6]. However, as a common arrhythmia drug, long-term use may cause adverse reactions in the cardiovascular system. Meanwhile, achieving effective drug concentrations in the wound area can be challenging due to drug metabolism and clearance following systemic administration [10]. Considering these limitations, localized delivery of propranolol may prove to be more effective for treating burn wounds.

Hydrogels are vital for dermal and epidermal regeneration, as they are capable of donating or absorbing water based on wound needs. Since their debut as wound dressings in 1977, hydrogels have advanced from single-component materials to complex formulations with nanoparticles, peptides, and growth factors [[11], [12], [13], [14]]. Gelatin and alginate have been examined for wound healing, with gelatin enhancing inflammation and healing through macrophage modulation and arginine-glycine-aspartic acid (RGD) sequences, while sodium alginate supports autolytic debridement [15,16]. Additionally, burn wounds are particularly susceptible to invasive infections from microorganisms before full epithelial regeneration. Serious infections can give rise to life-threatening complications such as sepsis, underscoring the necessity for immediate antimicrobial intervention [17]. Despite the recent development of various types of wound dressings for burns that significantly promote healing, the majority lack strong antibacterial properties to prevent infection in burn wounds and are ineffective against already infected wounds [[18], [19], [20]]. From the late 1990s onwards, studies have highlighted the crucial involvement of metal ions like magnesium (Mg^2+^), zinc (Zn^2+^), and copper (Cu^2+^) in tissue regeneration and remodeling processes [7,21]. Copper ions, in particular, have garnered considerable interest for their dual roles in skin regeneration and antibacterial activity. As a vital trace element, copper serves multiple physiological functions in human health including angiogenesis, collagen synthesis, and matrix remodeling, which are crucial across various wound healing stages [22,23]. Moreover, copper ions can destroy bacteria by disrupting their central metabolic and biosynthetic processes, exhibiting lower toxicity than silver, and are more viable for clinical use due to their abundance, availability, and cost-effectiveness [23,24].

In this study, we synthesized a multifunctional hydrogel by combining gelatin with alginate and incorporating the β-blocker propranolol. Copper ions, used as cross-linking agents in the hydrogel, provided sustained antimicrobial effects. This injectable anti-sympathetic hydrogel could reverse the negative effects of norepinephrine (NE) on human vascular endothelial cells (HUVECs). We also investigated the impact of the hydrogel on PC12 cells simulating the sympathetic nervous system in vitro. In animal experiments, the hydrogel not only exhibited antibacterial properties and promoted angiogenesis but also delayed the early innervation of the sympathetic nerves, successfully treating third-degree burns in SD rats.

## 2 Material and methods

### 2.1 Preparation of PNL@GA-Cu hydrogel

At a constant temperature of 60 °C, 6 g of gelatin (NeoFroxx, 1042GR100, Type B from Bovine Skin, Einhausen, Germany) and 2 g of sodium alginate (MW = 198, Solarbio, A9640, Beijing, China) were dissolved in 100 mL of deionized water with stirring 2 h to form a composite solution. Under magnetic stirring, propranolol hydrochloride (Aladdin, 318-98-9, Shanghai, China) was slowly added to the composite gel solution at concentrations determined by previous studies [10]. The propranol-containing composite solution was poured into molds or applied to wound tissues and allowed to cool at room temperature to form initial hydrogels. Subsequently, a 1.5 % (w/v) CuSO_4_ (Aladdin, 7758-98-7, Shanghai, China) solution was prepared using deionized water and added to the molds to allow copper ions to chelate with carboxyl groups in sodium alginate, forming a composite gel within 5 min. This process yielded PNL@GA-Cu hydrogels with a gelatin/alginate/propranolol ratio of 6:2:0.1 (w/w). All samples were washed in PBS (Biosharp, BL302A, Anhui, China) for 30 s to eliminate excess copper and unloaded propranolol. Similarly, a 1.5 % (w/v) CaCl_2_ (Aladdin,10043-52-4, Shanghai, China) solution was added to the initial hydrogel without propranolol and crosslinked for 5 min to obtain GA-Ca hydrogels.

### 2.2 Morphological characterization

The hydrogel was first freeze-dried and cut in the middle, followed by gold sputter coating to examine its internal morphology. The samples' morphological characteristics were then analyzed using a scanning electron microscope (SEM) (SEM 450; FEI, Hillsboro, USA).

### 2.3 Rheological properties of the hydrogels

The rheological properties of the hydrogels were assessed using a Kinexus rheometer (Malvern, Worcestershire, UK). Frequency scans from 0.1 to 10 Hz were performed for various hydrogel groups, and measurements of the storage modulus (G′) and loss modulus (G″) were taken to determine their rheological behavior.

### 2.4 Swelling capacity and biodegradation rate measurement

To assess the swelling capacity and degradation characteristics of the hydrogels, samples (n = 3) were air-dried at ambient temperature, and their dried weight was recorded as W_dry_. These samples were then immersed in PBS at 37 °C to reach swelling equilibrium, with their weight (W_wet_) recorded at predetermined intervals until the maximum weight (W_max_) was achieved. After reaching this point, the swollen hydrogels were continuously immersed with gentle stirring, and their weight (W_t_) was recorded over 14 days.

$$Swelling\;capacity\left(\%\right)=\frac{W_{wet}-W_{dry}}{W_{dry}}\times 100\%$$

$$Degradation\;rate\left(\%\right)=\frac{W_{\max}-W_{t}}{W_{\max}}\times 100\%$$

### 2.5 Drug release analysis

To study the drug release behavior, 200 μL of hydrogels were placed in 2 mL of PBS solution and incubated at 37 °C with gentle agitation. At predetermined intervals (6, 12, and 24 h; 2, 4, and 7 days), all solutions containing the released Cu^2+^ and PNL were collected and replenished with an equal volume of fresh PBS. The release of PNL was measured at 289 nm using an ultraviolet–visible spectrophotometer (UV-3101PC, Shimadzu, Japan), while the release of copper ions was determined by using an inductively coupled plasma source spectrometer (Prodigy-ICP, Leeman Labs, USA).

### 2.6 Preparation of hydrogel scaffold extracts

The scaffolds were thinned out using a DMEM F-12 cell culture medium (Gibco, 21331020,Grand Island, NY, USA) at a concentration of 20 mg/mL. They were left in an incubator set at 37 °C for 24 h. Using a pH meter, the pH levels of the extracts were registered. Standard sterilization was conducted on all the samples before usage.

### 2.7 Antibacterial activity test against *E. Coli* and *S. Aureus* of hydrogel

To evaluate the antibacterial efficacy of the GA-Ca, GA-Cu, and PNL@GA-Cu hydrogels, *Escherichia coli* (*E. coli*) and *Staphylococcus aureus* (*S. aureus*) strains obtained from the China Center for Type Culture Collection (Wuhan, China) were used. The bacterial strains were cultivated in LB broth at 37 °C. For the antibacterial assessment, 100 μL bacterial suspension (10^8 CFU/mL) was applied to the equal volume of different hydrogel scaffolds and incubated for 6 h. Ultrasound was used to detach the bacteria adhered to the hydrogels, and the resulting bacterial suspension was spread on agar plates. After incubating at 37 °C for 24 h, colony counting was performed to calculate the antibacterial efficiency.

Live/dead bacterial staining was conducted by incubating bacterial suspensions with a commercial Live/Dead bacterial staining solution (Maokang Biotechnology Co., Ltd., Shanghai, China), and the stained samples were imaged using a fluorescence microscope (IX73, Olympus, Japan). After fixation and dehydration, the bacteria were suspended in absolute ethanol, and a 100 μL aliquot was deposited onto a silicon wafer. The samples were then dried, gold-coated via sputter-coating, and visualized using a scanning electron microscope (SEM450; FEI, Hillsboro, USA).

### 2.8 Adhesive property

The stickiness of the hydrogel before crosslinking was evaluated by attaching the substance to numerous materials like plastic, the lung, heart, liver, spleen, and skin.

### 2.9 Biocompatibility testing

A Live/Dead assay was conducted to evaluate cell viability using a Live/Dead Cell Assay Kit (Solarbio, CA1630, Beijing, China). HUVECs (Procell, CL-0675, Wuhan, China) and PC12 cells (High differentiation, Stem Recell, STM-CL-7012, Shanghai, China) were cultured in 24-well plates. At predetermined time points, the assay was performed following the manufacturer's protocol. The stained samples (n = 3) were then examined under a fluorescence microscope.

The CCK-8 assay (Biosharp, BS350A, Hefei, China) was utilized to evaluate the cell proliferation of HUVECs and PC12 cells after being exposed to different hydrogel extracts. The cells were seeded at the same density per well within a 96-well plate (Corning, NY, USA). Following a 24-h incubation period, cells were gently washed with PBS twice and subsequently treated with GA-Ca, GA-Cu, or PNL@GA-Cu hydrogel extracts. After the cells were cultured for an additional 24, 48, or 72 h, the CCK-8 assay was executed following the instructions provided by the manufacturer.

To observe the morphology of cells growing on hydrogels, different hydrogels were evenly distributed in a 24-well plate, and HUVEC were seeded. After incubating for 24 h, actin staining was performed to visualize the cytoskeleton.

### 2.10 Hemolysis assay

We evaluated the hemocompatibility of the formulated hydrogels by hemolysis assay as previously reported [25]. Initially, red blood cells (RBCs) were collected from fresh blood of healthy SD rats via centrifugation at 2000 rpm for 10 min. Then, 100 μL of various hydrogels, 0.9 % NaCl (negative control), and distilled water (positive control) were incubated with a 500 μL RBC suspension at 37 °C for an hour. Afterward, the supernatants were collected through centrifugation, and their absorbance at 541 nm was measured using UV–Vis spectroscopy (Specord 210 Plus, Analytik Jena AG, Jena, Germany).

### 2.11 In vitro cell migration assay

The impact of scaffolds on HUVECs or PC12 cell migration was studied via a scratch wound healing assay (n = 3). HUVECs or PC12 were placed into 6-well culture plates (Corning, NY, USA) until approximately 90 % confluence was reached. A sterile 200 μL pipette tip was utilized to create wounds across each well, followed by a replacement of the hydrogel extracts with or without norepinephrine (1 μM) (MCE, HY-13715, NJ, USA). The cells were inspected and imaged at the start and after 24 h using an inverted microscope (IX73, Olympus, Japan). The wound closure area concerning the initial area was quantified with the help of ImageJ software.

A transwell migration assay was conducted to further validate the influence of hydrogels on cells (n = 3). In brief, a cell suspension of 200 μL in serum-free DMED-F12 was applied to the upper chamber, while the lower chamber was supplemented with 600 μL of serum-free scaffold extract mixed with or without norepinephrine (1 μM). At the 24-h mark, cells that migrated were stabilized using 4 % paraformaldehyde for 30 min. Post-removal of non-migrated cells in the inserts using a cotton swab, each insert was treated with 1 % (w/v) crystal violet for 10 min. Images of the cells in the lower chamber were captured using an inverted microscope. To gauge cell migration, the crystal violet was dissolved in 10 % acetic acid for 10 min and its absorbance was recorded using a microplate reader at 590 nm. The amount of cell migration was determined by the OD values.

### 2.12 Tube formation assay

The angiogenic capabilities of various scaffolds were examined in vitro using a Matrigel-based tube formation test (Corning, 356234, NY, USA) involving HUVECs. In brief, a 96-well plate was coated with 70 μL of Matrigel and incubated at 37 °C for 30 min to facilitate gel formation. Each well was seeded with 2 × 10^4^ HUVECs in varied extract media, with or without norepinephrine (1 μM). Following a 6-h incubation period, random snapshots were captured to evaluate the tubular network formation. For analysis purposes, Image J software was used to count the total segmental length and junction numbers (n = 3).

### 2.13 Western blotting

Western blot analysis was utilized to quantify the protein expression levels of angiogenic markers. HUVECs were cultured with various scaffold extracts in the presence or absence of norepinephrine (1 μM) over 5 days. Following culture, HUVECs were lysed with 100 μL of RIPA buffer, and after centrifugation, the supernatant was collected for protein quantification using a BCA kit (Beyotime Biotechnology, P0012, Shanghai, China) as per the manufacturer's protocol. The protein samples were then separated by SDS-PAGE and transferred onto a polyvinylidene difluoride (PVDF) membrane (Biosharp, BS-PVDF-45,Hefei, China). The membrane was blocked with 5 % skimmed milk (Biosharp, BS102,Hefei, China) for 1 h and subsequently incubated overnight at 4 °C with primary antibodies (Abcam, ab76533, anti-CD31, 1:5000; ab214424, anti-VEGF, 1:1000). After three washes with Tris-HCl and Tween (TBST) buffer, the membrane was exposed to secondary antibodies (Abcam, ab6721, 1:2000) for 30 min. Finally, chemiluminescence reagents (Biosharp, BL523A,Hefei, China) were used to visualize the protein bands, and the intensities of the bands were quantified using ImageJ software.

### 2.14 Animal experiment

Sprague-Dawley (SD) rats utilized in the study were obtained from Hubei Biont Biotechnology Co., Ltd, and all procedures were approved by the animal research ethics committee of Tongji Medical College, Huazhong University of Science and Technology. The procedure to establish infected burn wounds in rats was consistent with the previous study [2]. Briefly, male Sprague-Dawley rats weighing 180–200 g were anesthetized and removed hair on their back. A third-degree full thickness burn wound was created by a temperature control burn instrument with a 500 g preheated copper block at the temperature of 100 °C for 15 s at the dorsal region. Post-debridement, *S. aureus* was placed on the wound, followed by the application of either saline (Control group) or hydrogel. The burn wound was subsequently covered with a Tegaderm™ film dressing (3M Deutschland GmbH, Neuss, Germany). The images of wound healing regions were recorded on days 0, 1, 3, 7, and 14, and tissue samples were collected after 0, 1, 7, and 14-days post-surgery for H&E staining, Masson's trichrome staining, and immunofluorescent analysis (Abcam, ab7817, anti-α-SMA,1:100; ab182981, anti-CD31, 1:100; ab137869, anti-TH, 1:100, UK). Using the same exposure time and laser intensity for photographing each marker. The relative fluorescence intensity is obtained by comparing it with the fluorescence intensity of the control group.

### 2.15 ELISA

Plasma samples were prepared by centrifuging whole blood at 4 °C (1400×*g* for 10 min) and stored at −80 °C. The concentrations of norepinephrine and epinephrine in the plasma were measured using commercial ELISA kits (Cloud-Clone Corp., CEA907Ge/CEA858Ge, Houston, TX, USA). The assay was conducted according to the manufacturer's instructions, and the absorbance of the plates was read at 450 nm wavelength.

PC12 cells were incubated with Earle's balanced salt solution (EBSS) for 1 h, followed by EBSS containing 80 mmol L⁻^1^ KCl and different scaffolds for 30 min. Cells were then digested with trypsin and centrifuged at 1000×*g* for 5 min. The collected cells were washed three times with pre-chilled PBS, resuspended in PBS (1 × 10⁶ cells/mL), and disrupted by sonication. Finally, the cell suspension was centrifuged at 1500×*g* for 10 min at 2–8 °C, and the supernatant was collected. Tyrosine hydroxylase levels were measured using a commercial ELISA kit (Cloud-Clone Corp., SEB438Ra, Houston, TX, USA) according to the manufacturer's instructions.

### 2.16 Immunofluorescence staining

HUVEC and PC12 cells treated with hydrogels were fixed using paraformaldehyde. Subsequently, cells were permeabilized using a 0.1 % Triton X-100 PBS solution (Sigma, 9036-19-5, Darmstadt, Germany) and blocked with 0.5 % bovine serum albumin (Sigma, 10711454001, Darmstadt, Germany). Primary antibodies against CD31 and TH were then applied to the cells and allowed to incubate overnight at 4 °C. Following this, suitable secondary antibodies (Sigma, C2306, F1262, 1:200, USA) were applied and allowed to incubate for 1 h. Hoechst 33342 (Thermo fisher, 62249, Massachusetts, USA) for was used to nuclear staining and Dil (Thermo fisher, D282, Massachusetts, USA) for cell membrane staining. Additionally, FITC-NE (Qiyue Biology, Q-0128008, Xi'an, China) was used to evaluate the binding of NE to receptors on the cell membrane. Images were captured by using a fluorescent microscope.

### 2.17 Statistical analysis

All experimental data were analyzed using t-tests and one-way ANOVA performed with SPSS 26.0 software. Results are expressed as mean ± standard deviation. Statistical significance was considered at P < 0.05.

## 3 Results

### 3.1 Characterizations of PNL@GA-Cu antisympathetic hydrogel

**Fig. 1:**
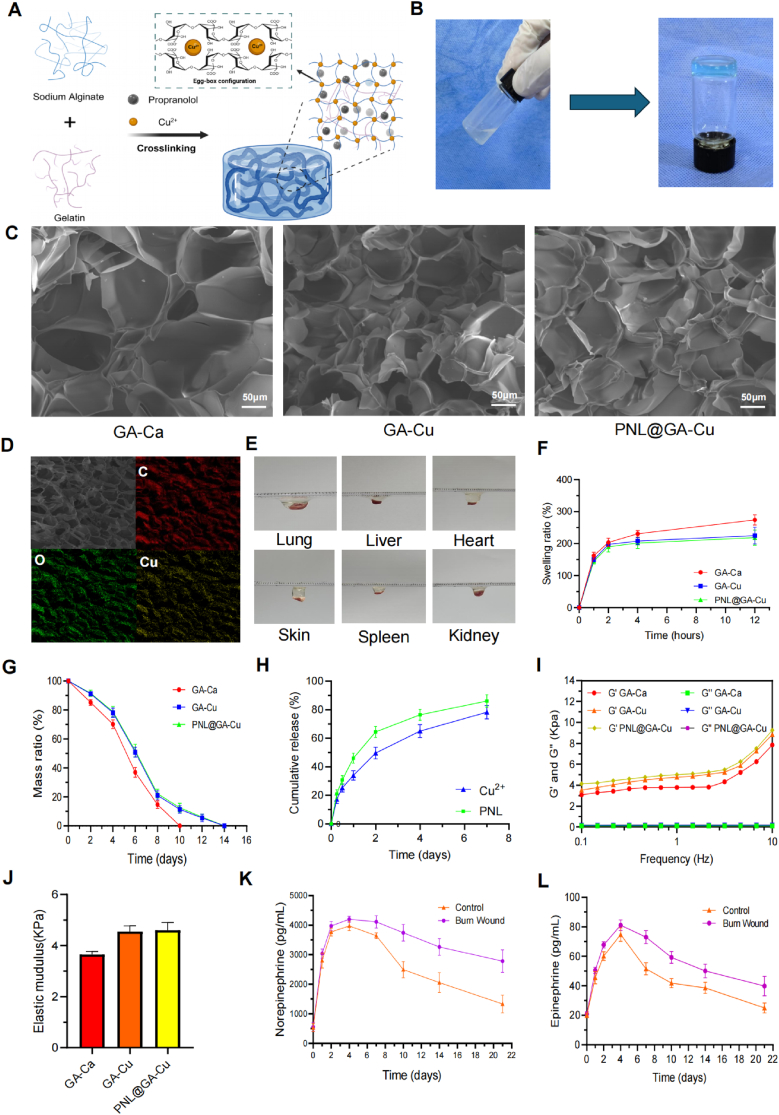
Hydrogel characterization and stress response in rats after burn injury. A) Schematic illustration of non-covalent crosslinking interaction in the hydrogel network; B) Solution of the PNL@GA and PNL@GA-Cu hydrogel; C) Magnified SEM images of the hydrogels; D) Elemental distribution mapping of PNL@GA-Cu hydrogel; E) Adhesiveness of the PNL@GA solution before crosslinking; F) Swelling ratio of GA-Ca, GA-Cu, and PNL@GA-Cu hydrogels in PBS (n = 3); G) Degradation and H) drug release behavior of the hydrogels in vitro (n = 3); I) The dynamic frequency sweep of GA-Ca GA-Cu, and PNL@GA-Cu hydrogels; J) Elastic modulus of hydrogels (n = 3); K-L) Impact of surgical trauma and burn injury on plasma levels of norepinephrine and epinephrine (n = 3); Created with BioRender.com.

In the preliminary study, we measured catecholamine levels in rat blood based on the previously reported theory that burn wounds might cause sustained sympathetic excitation, to confirm the necessity of loading propranolol into hydrogels [6]. The results (Fig. 1K and L) showed that both the burn injury group and the control group (surgery-related trauma) exhibited similar trends in blood norepinephrine and epinephrine concentration, which rapidly increased and peaked on the fourth day post-surgery, then gradually decreased during the wound healing process. Notably, catecholamine levels in the burn injury group remained consistently higher and declined more slowly than in the control group. By day 21 post-surgery, catecholamine levels in the control group had nearly returned to pre-surgery levels, whereas levels in the burn group remained elevated. These data demonstrate that chronic stress induced by burn injury can persist throughout the entire wound-healing process.

Alginate-based hydrogels typically use Ca^2^⁺ for crosslinking, but their affinity is weaker compared to other divalent ions such as Cu^2^⁺, Ba^2^⁺, and Sr^2^⁺, etc which directly affect the mechanical strength and gelation time [26]. Additionally, Ca^2^⁺ exhibits limited bioactivity towards endothelial cells, keratinocytes, and fibroblasts, restricting the application of alginate-based hydrogels in wound repair. Therefore, this study selects Ca^2^⁺ as a counterpart of Cu^2^⁺ to investigate their differences in hydrogel construction and biological activity. Due to the cytotoxicity of high concentrations of copper ions, we performed a CCK-8 assay to screen the concentration of CuSO_4_ used for hydrogel cross-linking. We ultimately determined that the optimal concentration of CuSO_4_ is 1.5 % (w/v) (Fig. S1). Fig. 1A illustrates the schematic of the PNL@GA-Cu hydrogel gelation. Throughout the process, the gelatin component in the hydrogel enhances its mechanical properties and drug-loading capacity. The Cu^2^⁺ replaces the sodium ions in the glucuronic acid monomer of sodium alginate, forming intermolecular bonds between Cu^2^⁺ and alginate chains and creating a linear, tightly bound “egg-box structure”. The FTIR spectra showed a blue shift in the absorption peaks of GA-Cu and PNL@GA-Cu samples compared to the Alg sample in the 1600–1700 cm⁻^1^ region (Fig. S2). This shift is likely due to an enhanced electron-withdrawing effect on the carbonyl groups caused by coordination, leading to an increased C=O stretching vibration frequency. This observation further indicates an interaction between Cu^2^⁺ ions and sodium alginate. As shown in Fig. 1B, during the preparation of the PNL@GA-Cu hydrogel, propranolol was thoroughly mixed with the gelatin and sodium alginate solution before gelation. The resulting hydrogel exhibited a light blue color due to the presence of Cu (Ⅱ).

The microporous structure and bioactive elements of the hydrogel are fundamental to its regeneration property. SEM images of three types of hydrogels (GA-Ca, GA-Cu, and PNL@GA-Cu) are shown in Fig. 1C. The morphological images of all the hydrogels revealed a highly porous, sponge-like structure. Notably, the pore size of the copper ion cross-linked hydrogels (30–100 μm) was smaller than that of the calcium ion cross-linked hydrogels (100–200 μm), enhancing the porosity of the hydrogels. Additionally, the incorporation of propranolol did not affect the pore size of the hydrogels. The internal porous structure of the hydrogel facilitates the formation of new tissues by meeting the needs of cell adhesion, migration, and nutrient supply [1,27]. Furthermore, carbon and oxygen are the basic elements of the hydrogel framework and its loaded propranolol, while Cu is the key element for promoting hydrogel gelation. Fig. 1D shows that, in the freeze-dried PNL@GA hydrogel, these elements are abundant and uniformly distributed throughout the hydrogel. In Fig. 1E, the PNL@GA solution can adhere to both inorganic and organic surfaces, such as plastic, lung, heart, liver, spleen, and skin, before gelation. This enables the constructed PNL@GA-Cu hydrogel to closely conform to the skin and form personalized wound shapes, demonstrating wide-ranging applications.

To assess the degradation and swelling properties of the hydrogel, dried samples were immersed in PBS and weighed at various time intervals. Fig. 1F shows the equilibrium swelling ratios of the hydrogels. The GA-Ca hydrogel demonstrated a swelling ratio of 273.67 ± 15.95 %, surpassing the GA-Cu hydrogel at 224.67 ± 26.63 % and the PNL@GA-Cu hydrogel at 218.33 ± 23.71 %. In addition, all hydrogels degraded rapidly (Fig. 1G). Notably, the GA-Ca hydrogel almost completely degraded within 10 days, whereas the degradation of GA-Cu and PNL@GA-Cu hydrogels was relatively slower, extending to 14 days may be due to more stable metal-ligand bond of Cu^2^⁺ compared to Ca^2^⁺ with larger bond strength and a longer bond lifetime [28]. Fig. S3 shows GA-Ca and GA-Cu hydrogels on the sixth day of degradation. Both hydrogels exhibited varying degrees of degradation, but the structure of the calcium-crosslinked hydrogel was compromised, while the copper ion cross-linked hydrogel maintained its initial shape. This indicates that copper ions enhanced structural stability and delayed degradation. The rapid degradation behavior indicates that the hydrogel can release Cu^2+^ and propranolol during the early stages of burn wound healing (Fig. 1H). In the PNL@GA-Cu hydrogel, the cumulative release of propranolol reached 46.06 ± 3.49 % on the first day and 86.09 ± 4.45 % on the seventh day. The release behavior of Cu^2+^ was very similar to that of propranolol, reaching 78.30 ± 4.65 % on the seventh day.

The rheological characteristics of the three hydrogels are depicted in Fig. 1I and J. All hydrogels demonstrated a storage modulus (G′) notably greater than the loss modulus (G″), suggesting the formation of robust hydrogel networks. Moreover, the elastic modulus of GA-Cu and PNL@GA-Cu hydrogels surpassed that of GA-Ca hydrogels, likely attributed to the enhanced binding capacity of copper ions, which contributes to the mechanical strength of the hydrogels.

### 3.2 In vitro antibacterial activity of hydrogels

**Fig. 2:**
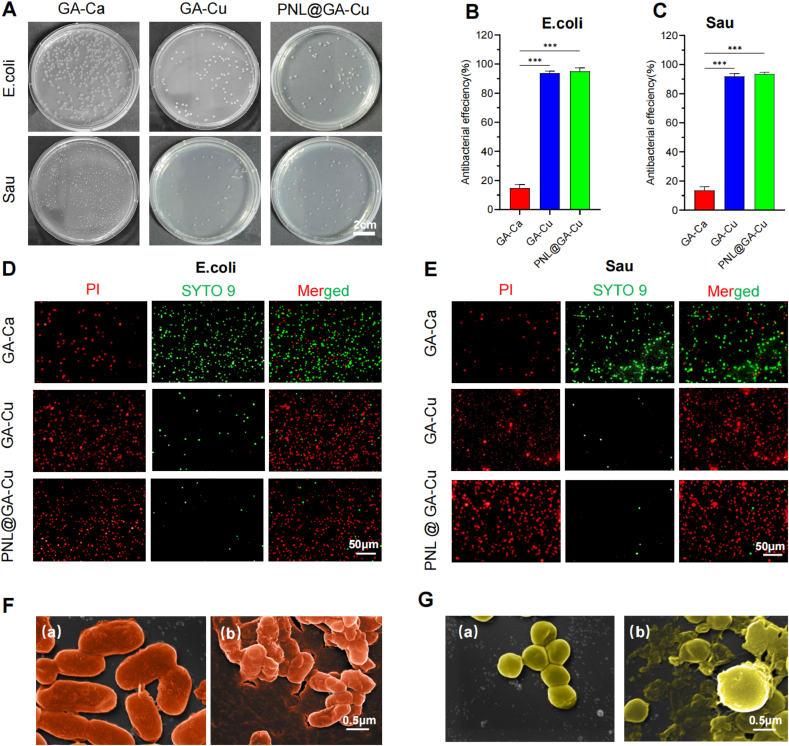
In vitro assessment of antibacterial effectiveness of hydrogels. A) Images of bacterial colonies grown on agar plates across various treatment groups; Quantification of colony numbers in (A) shows the antibacterial effectiveness of various hydrogels against B) *E. coli* and C) *S. aureus* (n = 3); Representative images of bacterial Live/dead staining for D) *E. coli* and E) *S. aureus* exposed to different hydrogels; Magnified SEM images of F) *E. coli* and G) *S. aureus* after treatment with (a) control and (b) PNL@GA-Cu hydrogel; Statistical significance denoted as ∗P < 0.05, ∗∗P < 0.01, ∗∗∗P < 0.001.

The in vitro antibacterial efficacy of the hydrogels was assessed against *E. coli* and *S. aureus*. Fig. 2A–C illustrates the initial assessment of antibacterial effectiveness performed with the spread plate method. Calcium ion cross-linked hydrogels did not exhibit notable antibacterial activity. In contrast, hydrogels with copper ions maintained their antibacterial properties irrespective of the inclusion of propranolol. After 24 h of incubation, the antibacterial efficiencies of the GA-Ca, GA-Cu, and PNL@GA-Cu hydrogels against *E. coli* were 14.78 ± 2.42 %, 93.85 ± 1.40 %, 95.11 ± 2.24 %, respectively. Against *S. aureus*, these efficiencies were 13.48 ± 2.59 %, 91.95 ± 2.01 %, and 93.55 ± 1.21 %, respectively. In Fig. 2D and E, bacteria were assessed using live/dead staining, where live bacteria emit green fluorescence and dead bacteria emit red fluorescence. In the GA-Ca group, predominantly green fluorescence was observed with few instances of red fluorescence, indicating limited antibacterial activity. In contrast, the GA-Cu and PNL@GA-Cu hydrogels exhibited robust antibacterial effects, as evidenced by prominent red fluorescence and minimal green fluorescence. These results align with findings from the spread plate method, confirming the hydrogels' antibacterial efficacy. In Fig. 4F and G, SEM images illustrate the morphologies of *E. coli* and *S. aureus* following exposure to the hydrogels. In the control group, both bacterial species retained their typical shapes without membrane damage. Conversely, bacteria exposed to PNL@GA-Cu hydrogels showed significant damage, characterized by irregular shapes, wrinkled membranes and contents leaking out.

### 3.3 Biocompatibility of the anti-sympathetic hydrogel

**Fig. 3:**
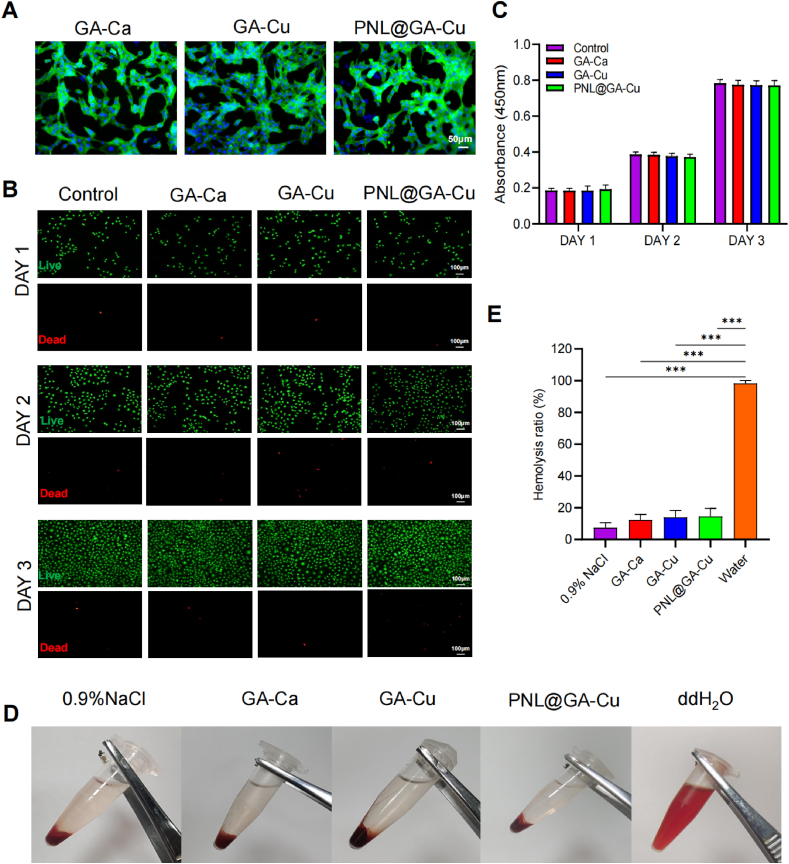
Biocompatibility assessment of the various hydrogels. A) Images of HUVEC growth on various hydrogels stained with phalloidin (green) and Hoechst (blue); B) Evaluation of HUVEC viability treated with different hydrogels using live/dead staining; C) CCK-8 assay to assess HUVEC viability following treatment with various hydrogels (n = 3); D-E) Hemolysis test of GA-Ca, GA-Cu, and PNL@GA-Cu hydrogels (n = 3). Statistical significance denoted as ∗P < 0.05, ∗∗P < 0.01, ∗∗∗P < 0.001. (For interpretation of the references to colour in this figure legend, the reader is referred to the Web version of this article.)

First, the biocompatibility of the hydrogel was assessed using phalloidin staining of HUVECs inoculated on the hydrogel (Fig. 3A). The HUVECs exhibited a typical elongated, spindle-like morphology, indicating that the hydrogel supports the normal shape of endothelial cells with minimal harmful stimuli. The cell density on the hydrogel was high and uniformly distributed, reflecting good cell proliferation and effective support for cell growth. Phalloidin staining showed bright and continuous actin filaments, highlighting live and healthy cells. The absence of fragmented or faint staining further confirms the hydrogel's biocompatibility, as cells remained viable and healthy throughout the incubation period. Furthermore, live/dead staining was employed to assess the cytocompatibility of the hydrogels with HUVECs (Fig. 3B). The results indicated that all the hydrogels were non-toxic to HUVECs, demonstrating low cytotoxicity. The CCK-8 assay results also indicated that the proliferation rate of HUVECs in the PNL@GA-Cu group was comparable to that in the GA-Ca, GA-Cu, and control groups (Fig. 3C). This suggests that the PNL@GA-Cu hydrogel does not negatively impact cell growth and maintains similar levels of cell proliferation as the other groups tested. As demonstrated in Fig. 3D and E, the hemolysis rates for the control group, GA-Ca, GA-Cu, and PNL@GA-Cu were significantly lower than those observed in the water group. These results highlight the excellent blood compatibility of the developed hydrogels. Additionally, we performed H&E staining on the hearts, livers, spleens, lungs, and kidneys of SD rats implanted with PNL@GA-Cu and untreated controls, revealing no significant differences between the two groups (Fig. S4). Such findings are crucial as they demonstrate that the PNL@GA-Cu hydrogel can support cellular activities essential for tissue regeneration and repair, making it a promising candidate for burn wound healing.

**Fig. 4:**
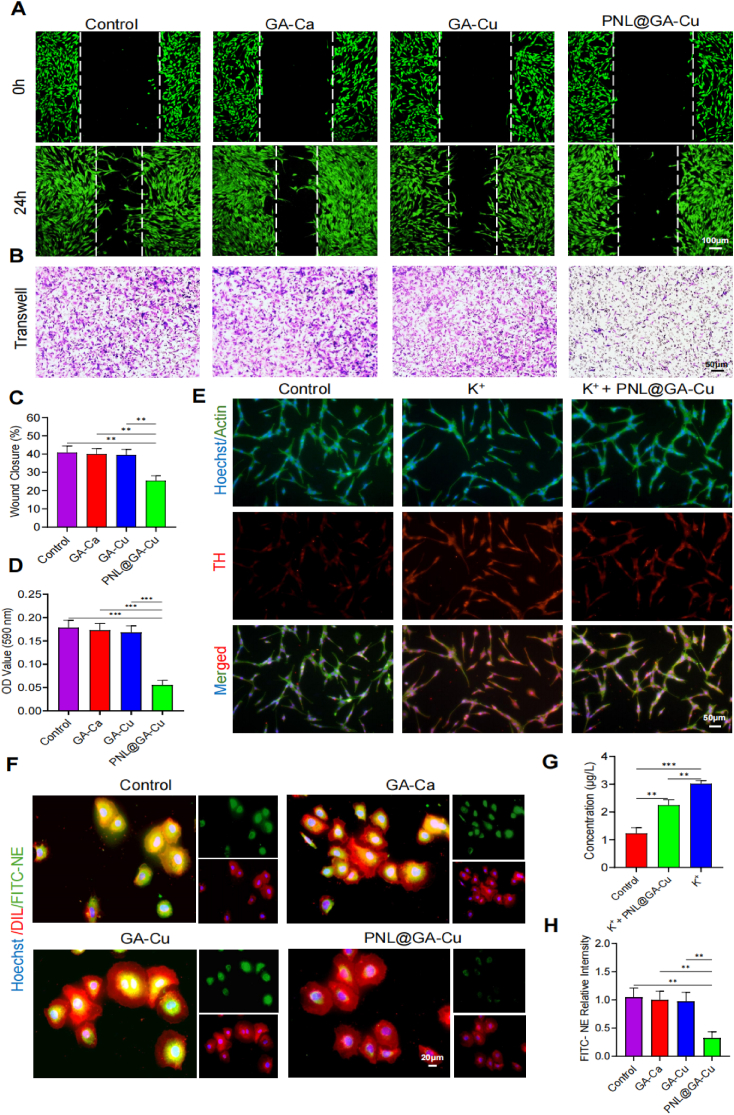
Hydrogels influence sympathetic nerve activity in vitro. A) Representative images of wound healing in PC12 cells stained with calcein-AM; B) Transwell migration assay results for different groups at 24 h; C) Wound closure rate observed in the wound healing assay (n = 3); D) Semi-quantification of PC12 transwell assay (n = 3); E) Immunofluorescence staining of tyrosine hydroxylase in PC12 cells; F) Typical fluorescence images of HUVECs treated with FITC-NE and hydrogels; G) Quantification of tyrosine hydroxylase expression and H) efficiency of FITC-NE binding to cell membranes (n = 3). Blue: nucleus; Red: cell membranes; Green: FITC-NE. Statistical significance denoted as ∗P < 0.05, ∗∗P < 0.01, ∗∗∗P < 0.001. (For interpretation of the references to colour in this figure legend, the reader is referred to the Web version of this article.)

### 3.4 Hydrogel affects the activity of sympathetic nerves in vitro

During wound healing, excessive sympathetic nerve activity may impede the repair process [6,7]. We utilized PC12 cells to study the effects of the hydrogel on sympathetic nerve migration and activity in vitro. The effects of the hydrogels on PC12 cell migration were evaluated using scratch assays and transwell assays (Fig. 4A–D). The wound scratch assay revealed that the wound healing rate in the PNL@GA-Cu group (25.40 ± 2.71 %) was significantly slower compared to the control group (40.80 ± 3.60 %), as well as the GA-Ca group (39.97 ± 3.01 %) and the GA-Cu group (39.53 ± 3.07 %) (Fig. 4A–C). In addition, we also stained the cells with calcein-AM at the beginning of scratching and after migration. We found that PC12 cells on both sides of the scratch not only migrated and exhibited axonal growth after 24 h but also showed proliferative activity (Fig. 4A). The transwell assay was further used to verify the effect of the hydrogel on cell migration (Fig. 4B–D). The findings indicated that the quantity of migrating PC12 cells was notably reduced in the PNL@GA-Cu group compared to the other groups, likely attributable to the release of propranolol. Additionally, PC12 cells were exposed to the hydrogel extracts, followed by live/dead cell staining and CCK8 assays, which indicated that the viability of PC12 cells treated with the samples from each group was comparable (Figs. S5 and 6). Above all, these results indicate that the regulation of sympathetic nerves by hydrogel is mainly through cell migration rather than inhibition of proliferation.

The effects of the sympathetic nerves on their target tissues are mainly mediated by the neurotransmitters they secrete, particularly NE. Tyrosine hydroxylase (TH), the key enzyme in catecholamine synthesis within sympathetic nerves, plays a crucial role in regulating neurotransmitter secretion by these cells [29]. To investigate TH expression, we employed immunofluorescence staining and ELISA assay (Fig. 4E–G). Our results indicated that the control group exhibited the lowest relative TH expression. Potassium ion stimulation significantly enhanced TH expression, aligning with previous studies [30,31]. Notably, the addition of PNL@GA-Cu hydrogel resulted in a marked downregulation of TH expression. Furthermore, we used FITC-labelled norepinephrine (FITC-NE) to study the effect of the anti-sympathetic hydrogel on target tissues (Fig. 4F–H). Our results showed that in the control group, GA-Ca group, and GA-Cu group, there was strong green fluorescence on the cell membranes of HUVECs, indicating that NE can tightly bind to receptors on the cell membrane (red). However, in the PNL@GA-Cu group, the binding of NE (green) on the cell membrane was significantly reduced. This suggests that propranolol released from the hydrogel can effectively compete with NE in the microenvironment, providing a catecholamine protective effect on target cells such as HUVECs.

### 3.5 Anti-sympathetic hydrogel promotes angiogenesis in vitro

**Fig. 5:**
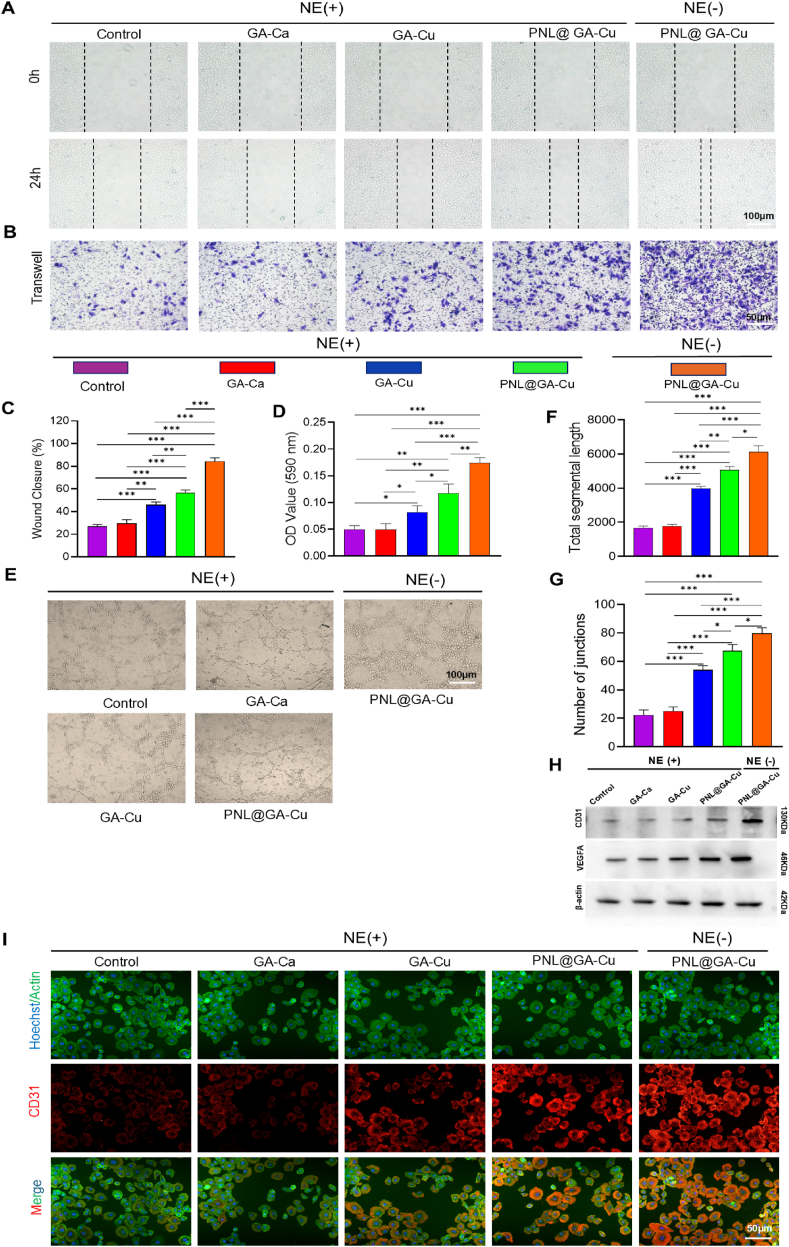
In vitro angiogenic ability evaluation of hydrogels. A) Representative images of HUVECs wound healing and B) transwell migration assay in various hydrogel treatment groups at 24 h; C) Wound closure rate of wound healing assay (n = 3); D) Optic density value of crystal violet dyes from transwell assay (n = 3); E) Tube-formation assay of HUVECs and semi-quantification analysis of F) total segments length and G) junction numbers in different groups (n = 3); H) Western blot detection of angiogenic protein in each group; I) Images of CD31 immunofluorescence staining in HUVECs cultured with various hydrogels. Statistical significance denoted as ∗P < 0.05, ∗∗P < 0.01, ∗∗∗P < 0.001. (For interpretation of the references to colour in this figure legend, the reader is referred to the Web version of this article.)

The recruitment of endothelial cells is vital for the formation of new blood vessels during skin repair, essential for restoring nutrient transport [11,13]. As illustrated in Fig. 5A, the cell scratching assay demonstrated that cell migration was significantly inhibited by NE. However, the migratory rates significantly improved after being treated with GA-Cu or PNL@GA-Cu hydrogel. The migratory ability was further analyzed based on the relative wound closure rate (Fig. 5C). After 24 h of incubation, the PNL@GA-Cu group without NE treatment exhibited the highest wound healing rate at 84.32 ± 3.17 %. Among the cells treated with NE, the release of propranolol in PNL@GA-Cu effectively counteracted the inhibitory effect of sympathetic nerve on the migration of HUVECs, resulting in a significantly higher migration ratio (56.75 ± 2.35 %) compared to the GA-Cu (45.93 ± 2.52 %), GA-Ca (29.68 ± 3.17 %) and control (27.13 ± 1.55 %) groups. Similarly, the transwell assay results further confirmed that the PNL@GA-Cu scaffold can significantly promote the migration of HUVECs under the inhibitory effect of NE (Fig. 5B). The number of HUVECs migrating to the lower chamber was markedly higher in the PNL@GA-Cu group with or without NE compared to the other groups (Fig. 5D).

The pro-angiogenic potential of the scaffolds was additionally assessed through a tube formation assay, which evaluated the stimulation of HUVEC migration in vitro. Enhanced formation of primary capillary-like networks was observed in the PNL@GA-Cu (NE-), PNL@GA-Cu (NE+), and GA-Cu (NE+) groups, whereas the GA-Ca (NE+) group and the control group showed minimal formation of tube-like structures (Fig. 5E). Further quantitative analysis using ImageJ software measured the total segmental length and the number of junctions after 6 h of incubation on Matrigel (Fig. 5F and G). There was no significant difference between the control group and the GA-Ca group in any of the indicators. In contrast, the GA-Cu (NE+), PNL@GA-Cu (NE+), and PNL@GA-Cu (NE-) groups showed significant improvements in these indicators (p < 0.01). It is worth noting that in PNL@GA-Cu (NE+) group, the total segmental length and the number of junctions reached 5079 ± 198.51 μm, and 67.33 ± 4.51, respectively, showing the suboptimal level among all the groups which is only lower than PNL@GA-Cu (NE-) group (6130 ± 358.6 μm and 77.33 ± 6.11). Similarly, Western blotting detection for angiogenic proteins such as VEGF and CD31 showed a similar trend to that of the tube formation assay (Fig. 5H). Moreover, we assessed the CD31 expression levels in HUVECs following various treatments using immunofluorescence staining (Fig. 5I). In the presence of NE, which simulates chronic sympathetic excitation, the expression of CD31 in HUVECs treated with GA-Cu and PNL@GA-Cu hydrogel was elevated, accompanied by stronger fluorescence intensity. Especially in the absence of NE, HUVECs treated with PNL@GA-Cu hydrogel showed the strongest CD31 expression. These results show that propranolol and copper ions released by scaffolds can promote cell migration and the formation of tubular networks under chronic stress-induced sympathetic excitation.

### 3.6 Anti-sympathetic hydrogel promotes infected burn wound healing

Burn wounds can be categorized as first-degree, second-degree superficial partial-thickness, second-degree deep partial-thickness, third-degree full-thickness, or fourth-degree full-thickness burns [1]. A first-degree burn affects only the epidermis and does not cause blistering. Superficial second-degree burns affect the papillary dermis, while deep partial-thickness burns extend into the reticular dermis. Both types typically manifest with blister formation occurring either immediately or within hours after injury [1,32]. In deep partial-thickness burns, unlike superficial second-degree burns, both blood vessels and nerve endings are damaged. Similarly, third-degree burns extend through the dermis and subdermal tissues, destroying blood vessels and nerve endings as well. Fourth-degree burns penetrate all skin layers and affect underlying muscles [1,32]. In this study, to confirm third-degree full-thickness burns, SD rats were sacrificed at 30 min and 24 h post-injury, followed by histological analysis of the burned tissue to assess burn depth (Fig. S7). The findings indicate that the morphological characteristics observed at both 30 min and 24 h post-injury are consistent, affirming the comprehensive impact of the burn on all three skin layers: the epidermis, dermis, and hypodermis. Destruction of all epidermal layers was evident. Furthermore, the dermis, encompassing both the papillary and reticular layers along with their vasculature, nerve endings, and collagen matrix, was entirely compromised. The hypodermis exhibited damaged adipocytes lacking nuclei, confirming the full-thickness nature of the burn injury. However, the underlying muscles, typically affected in fourth-degree burns, remained intact and showed no microscopic alterations.

**Fig. 6:**
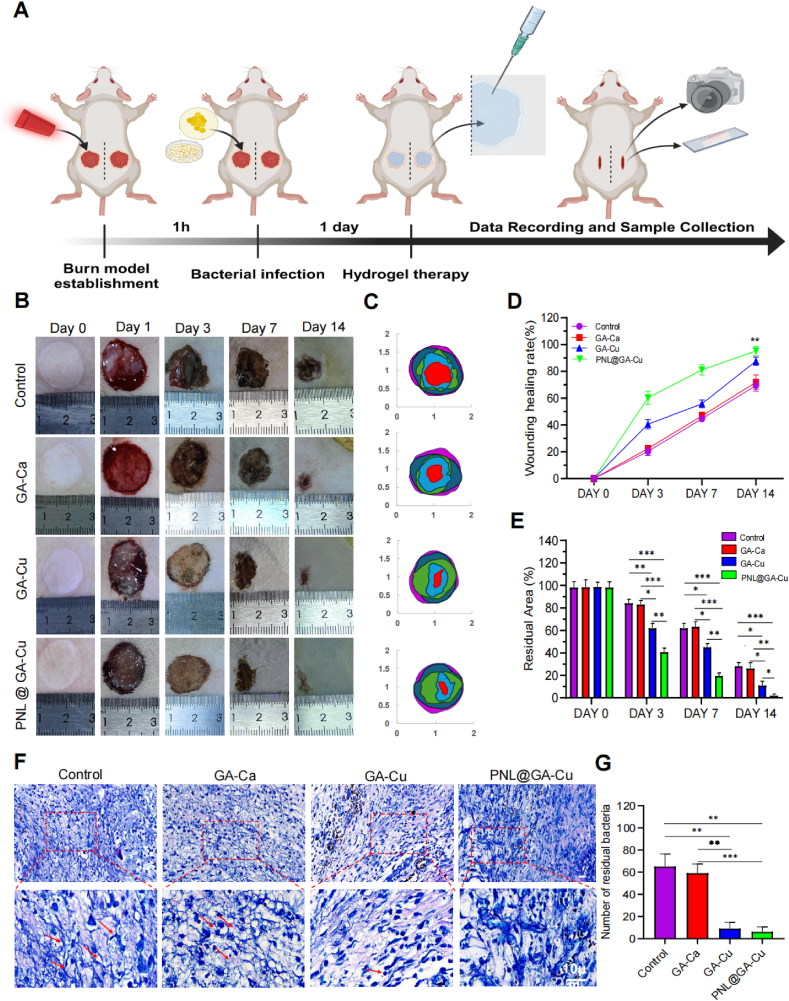
In vivo assessment of hydrogel effects on wound healing in rats with infected burn injury. A) Schematic diagram showing the prepared process of infected burn wound in Sprague-Dawley rats; B) Representative photograph of wounds across all groups at various stages of regeneration; C) The wound area at each time interval post-wounding is superimposed on the image; D) Quantification of wound closure rate and E) residual area (n = 3); F) Giemsa staining of wound tissues after 7 days of treatment. Red arrows indicate the presence of bacteria; G) Quantification of residual bacteria as shown in (F) (n = 3). Statistical significance denoted as ∗P < 0.05, ∗∗P < 0.01, ∗∗∗P < 0.001. Created with BioRender.com.(For interpretation of the references to colour in this figure legend, the reader is referred to the Web version of this article.)

We evaluated the wound healing process by measuring the wound size and calculating the healing rate. After 3 days of treatment, the PNL@GA-Cu hydrogel showed a significantly higher wound healing rate compared to the other groups **(p < 0.05,** Fig. 6B–D**)**. On Day 7, the wound closure rates were 44.63 ± 2.27 % for the control group (phosphate-buffered saline [PBS]), 46.90 ± 2.80 % for GA-Ca, 55.80 ± 2.92 % for GA-Cu, and 81.13 ± 3.79 % for PNL@GA-Cu. By Day 14, wounds treated with PNL@GA-Cu were nearly healed, with a closure rate of 95.30 ± 3.34 %, compared to 69.47 ± 4.21 % in the control group, 71.83 ± 5.50 % in the GA-Ca group, and 87.60 ± 3.11 % in the GA-Cu group. Over time, the PNL@GA-Cu group consistently exhibited a stable healing rate. Meanwhile, the remaining wound areas in the PNL@GA-Cu and GA-Cu groups were under 15 %, whereas those in the control and GA-Ca groups exceeded 25 % (Fig. 6E). The slow healing of burn wounds can be attributed to various pathophysiological mechanisms, including oxidative stress, fibroblast dysfunction, and impaired angiogenesis [3,33]. The control and GA-Ca groups lack sufficient bioactive components to counteract these adverse factors, resulting in less effective outcomes compared to the GA-Cu and PNL@GA-Cu groups.

Giemsa staining was utilized to evaluate the infection level in peri-implant tissues one week after surgery (Fig. 6F). The Giemsa-stained images of the control and GA-Ca groups revealed numerous bacteria (indicated by red arrows). In contrast, the GA-Cu and PNL@GA-Cu groups showed a significant reduction in bacterial numbers, indicating that the infection was effectively controlled (Fig. 6G). These results are consistent with the digital photographs shown in Fig. 6B. In the early stages of wound healing, the Control and GA-Ca groups exhibited signs of moisture and pus discharge, which may be associated with the delayed clearance of infection. The above findings demonstrate that the antibacterial properties of the GA-Cu and PNL@GA-Cu hydrogels effectively eradicated bacterial cells in the tissue due to the presence of copper ions, facilitating the repair of burn wounds.

**Fig. 7:**
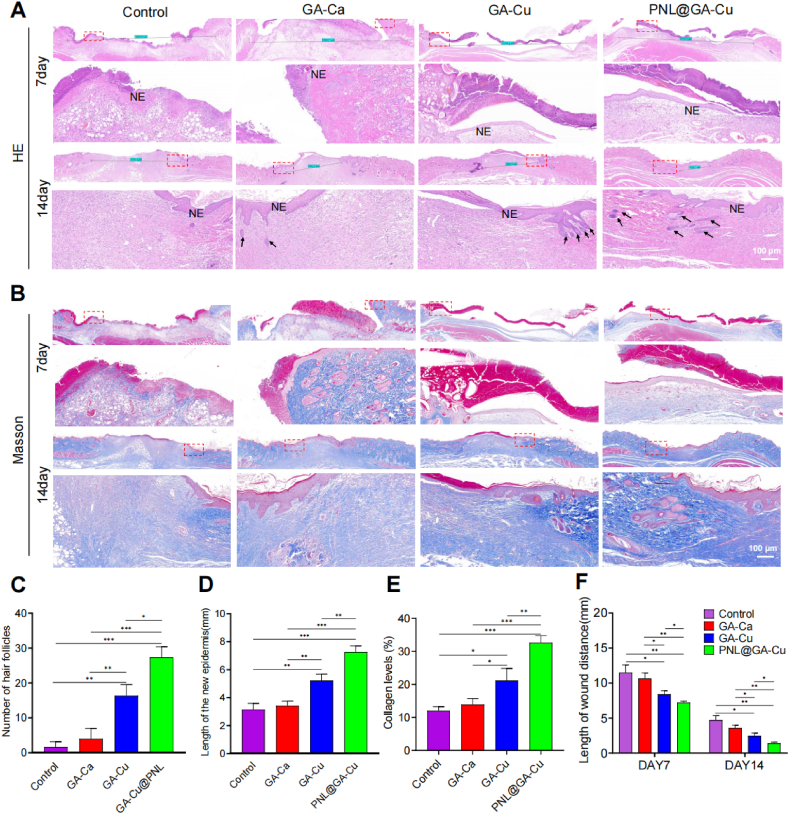
Histological staining analysis after hydrogel treatment. A) H&E and B) Masson's trichrome staining at 7- and 14-days; C–F) Statistical analysis of the new hair follicles, new epidermis length, collagen levels and wound distance (n = 3). Statistical significance denoted as ∗P < 0.05, ∗∗P < 0.01, ∗∗∗P < 0.001.

H&E and Masson's trichrome staining were also conducted (Fig. 7A and B). The PNL@GA-Cu treated groups more frequently exhibited typical characteristics of mature skin, including the formation of hair follicles and sebaceous glands (Fig. 7C). Additionally, the crawling distance of the newly formed epithelium was measured in the H&E-stained sections of the wounds, followed by a quantitative analysis of these measurements. As illustrated in Fig. 7A–D, and F, the PNL@GA-Cu treatment led to a longer crawling distance of the epidermis, indicating faster re-epithelialization compared to the control, GA-Ca, and GA-Cu groups. The blue colour in the Masson staining images represents newly formed collagen fibers, which are essential for the structure and function of skin mucosa and indicate tissue remodeling at the wound site. Consistent with the H&E staining results, Masson staining and quantitative collagen deposition analysis showed that the collagen deposition rate in the PNL@GA treatment group was significantly higher than in the other three groups at day 14 (Fig. 7B–E).

**Fig. 8:**
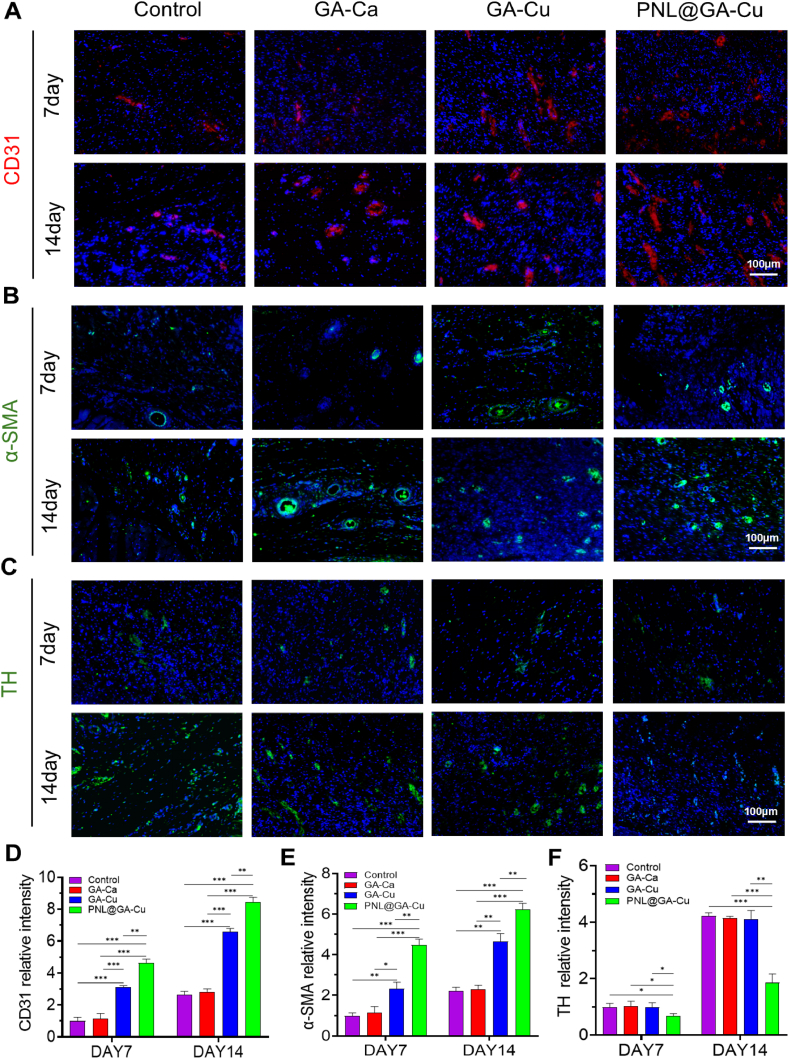
Effects of hydrogel on angiogenesis and sympathetic innervation in vivo. Immunofluorescence staining results of A) CD31, B) α-SMA, and C) TH at day 7 and day 14; D-F) Semi-quantification analysis of CD31, α-SMA and TH at day 7 and day 14 (n = 3). Statistical significance denoted as ∗P < 0.05, ∗∗P < 0.01, ∗∗∗P < 0.001.

Furthermore, immunofluorescence was used to assess the extent of revascularization and sympathetic nerve reinnervation at the repair site. As shown in Fig. 8A, the GA-Cu and PNL@GA-Cu groups exhibited more new blood vessels, with statistical analysis revealing significantly higher CD31 fluorescence density in these groups compared to the GA-Ca and control groups (Fig. 8D). The expression of α-SMA was also significantly increased after hydrogels containing Cu and Propranolol treatment, showing a similar trend to that observed for CD31 (Fig. 8B–E). An important observation is that the hydrogel incorporating propranolol slowed down the initial sympathetic reinnervation in newly formed skin. This was evidenced by a reduction in TH expression in tissues on day 7 and 14 (Fig. 8C–F).

## 4 Discussion

Mucosa, epidermis, and subcutaneous tissue harbor abundant sympathetic nerve fibers contributing to the maintenance of microenvironmental homeostasis [34]. However, studies have shown that sympathetic nerve activity negatively impacts wound healing by affecting critical processes such as the immune cells' response, keratinocyte migration, and fibroblast contraction [8,35]. When soft tissue is injured, stressors trigger the release of epinephrine and norepinephrine from the adrenal medulla and presynaptic neurons, which bind to β-AR on cell membranes [36]. Pathological changes resulting from β-AR activation can disrupt the wound-healing process. Particularly, severe thermal injury, surpassing other types of traumas in severity, leads to a series of pathological symptoms and triggers a hypermetabolic response, significantly increasing mortality rates [6]. In our initial exploration, catecholamine concentrations in rat blood increased rapidly after burn injury and remained elevated throughout the wound healing process, a finding consistent with previous studies (Fig. 1K and L) [6]. Catecholamines have long been recognized as crucial mediators of the hypermetabolic response following burns [9]. Extensive research has shown that counteracting the detrimental effects of elevated catecholamines can enhance the healing of acute wounds, chronic non-healing ulcers, severe thermal injuries, corneal wounds, and various other soft tissue disorders [8,[37], [38], [39], [40]].

Propranolol, a commonly used clinical beta-receptor-blocking agent, is primarily prescribed for cardiovascular diseases. It can counteract the harmful effects of catecholamines and has been used to reduce the elevated metabolic rates following burns, which are mediated by increased catecholamines or inflammatory mediators [6,9,41,42]. However, long-term use of β-receptor blockers like propranolol can lead to cardiovascular issues such as decreased blood pressure and bradycardia. Additionally, drug metabolism and clearance may prevent the medication from achieving effective concentrations at the defect site when administered systemically [10,43]. Currently, locally delivering drugs to counteract the heightened activity of sympathetic nerves after tissue injury has emerged as a promising strategy in tissue engineering [7,10,31,44]. Hydrogels are widely used in wound repair due to their excellent biocompatibility, degradability, and sustained drug-release properties [45]. In this study, we developed copper cross-linked gelatin-alginate hydrogels loaded with propranolol. This innovative scaffold demonstrated excellent mechanical properties and biocompatibility in both in vitro and in vivo. Notably, in vitro kinetic release assays revealed that the scaffold provided a sustained release of copper ions and propranolol for at least 14 days, suggesting that drug release could be maintained throughout the entire burn wound healing process.

Through the sustained release of copper ions and propranolol, the hydrogel eliminated most bacteria in the wound. Delayed wound healing is closely linked to persistent bacterial colonization and its harmful effects. Chronic wounds provide a favorable environment for bacterial growth, leading to complications such as infection and these bacteria form biofilms to evade host immune defenses [46,47]. Although antibiotics can be embedded in wound dressings to combat bacteria, their excessive use can lead to a significant increase in antibiotic-resistant bacteria. Biomaterials modified with copper ions present a novel infection prevention strategy, recognized by the Environmental Protection Agency (EPA) as an antimicrobial metal in 2008 [48]. In recent years, the properties and mechanisms of various copper-doped biomaterials have been extensively investigated by researchers [49]. In 2023, Xu et al. reported that copper ion-modified germanium phosphorus nanosheets can effectively kill *E. coli* and *S. aureus* and reduce bacterial activity [50]. Silicene nanosheets loaded with copper-containing nanoparticles demonstrate triple enzyme mimicry activities and a photothermal effect, aiding in the healing of bacteria-infected wounds [51]. The literature thoroughly documents the bactericidal mechanisms of copper, typically divided into three main stages: initially, copper ions are released, compromising the bacterial outer cell membrane and causing cytoplasmic leakage; subsequently, reactive oxygen species (ROS) are produced, inflicting additional bacterial damage; and finally, DNA rupture occurs, leading to bacterial death [52]. Additionally, the bacterial Quorum Sensing (QS) system detects adrenergic signals from the host's physiological state, prompting bacteria to increase motility, alter gene expression, and form biofilms, thereby enhancing their virulence [53]. Previous studies show that catecholamines increase *Pseudomonas aeruginosa* numbers after antibiotic treatment, which can be inhibited by β-blockers [54,55]. Therefore, β-blockers exert an anti-virulence effect by inhibiting the QS system, thereby helping to prevent infection and promote wound healing. While our results did not indicate the enhanced antibacterial performance of copper ions in synergy with propranolol, we still believe that loading propranolol inhibited biofilm formation and prevented infection recurrence.

Anti-sympathetic hydrogels exhibit enhanced vascular regeneration performance in the context of burn-induced stress. During regeneration, forming a new vascular system is essential for complete healing as it facilitates nutrient delivery, and oxygen supply, and establishes an inflammatory environment [56]. Studies have shown that mice with depleted NE levels exhibit enhanced wound angiogenesis and rats treated with β-AR antagonists display increased vascularization in skin wounds [57]. Further research has found that activation of β2-adrenergic receptors (β2-AR) inhibits endothelial cell mobilization, proliferation, and tube formation [58,59]. Additionally, recent studies have indicated that copper ions not only possess antimicrobial properties but also have a positive effect on angiogenesis by upregulating angiogenic genes such as VEGF and bFGF in both in vitro and in vivo settings [33,60]. Similarly, our results indicate that the sustained release of propranolol from the hydrogel can counteract the inhibition of angiogenesis by NE and synergize with copper ions, positively impacting early vascular regeneration in burn wounds (Fig. 5). This is consistent with previous studies showing that local delivery of propranolol helps maintain tissue homeostasis and enhances local angiogenic activity [31,61].

Compared to the other groups, the scaffold containing propranolol delayed early sympathetic innervation. PC12 cells, derived from the adrenal medulla of rats and able to exhibit an adrenergic phenotype following specific differentiation protocol as well as synthesize and store catecholamines, are often used to simulate sympathetic neurons [31,44]. In vitro experimental results showed that propranolol released from the hydrogel effectively reduced the migration rate of PC12 cells within 24 h without causing significant proliferation inhibition or cytotoxicity. Moreover, Tyrosine hydroxylase (TH) is the key enzyme in catecholamine neurotransmitter synthesis, and its activity is controlled by various mechanisms crucial for normal physiological functions [29]. Jia et al. found that Epinephrine elevated the expression of TH in PC12 cells in a dose- and time-dependent manner, an effect that was blocked by propranolol but not by phenoxybenzamine [62]. Long-term injection of propranolol was also found to reduce tyrosine hydroxylase and dopamine beta-hydroxylase activities in sympathetic ganglia [63]. These results align with our study, which demonstrates that high potassium levels enhance tyrosine hydroxylase expression, while propranolol released from hydrogel scaffolds significantly inhibits potassium-induced TH upregulation, which may reduce NE secretion by PC12 cells [30]. Notably, the sympathetic nerves activity in newly formed wound tissues in vivo mirrored the in vitro experiments, with propranolol reducing TH expression in local tissues on days 7 and 14. This is consistent with previous findings. Wu et al. found that the local release of propranolol via implanted 3D-printed scaffolds significantly delayed the ingrowth of TH + sympathetic nerves into the new bone defect areas over 4 weeks, with this effect becoming more pronounced with increased propranolol loading in the scaffolds [10]. We speculate that local β-receptor blockade may affect the production of chemotactic signals, such as semaphorin 3A (Sema3A), and inhibit the positive feedback effect of NE secreted by sympathetic nerves on the nerves themselves, thereby reducing sympathetic innervation and function [62,64].

However, this study has certain limitations. In vitro experiments suggest that propranolol scaffolds promote vascular formation during wound healing by competing with norepinephrine, indicating the need to investigate the role of exogenous norepinephrine in wound repair processes in vivo. Furthermore, quantifying the density of β-adrenergic receptors and catecholamine levels in the defect zone is essential for understanding the impact of propranolol on the sympathetic system within the scaffolds. Additionally, since the dual effects of propranolol in sympathetic nerve blockade and immune modulation, further research is needed to elucidate the changes occurring in the immune system within tissues treated with propranolol. Finally, given that sympathetic nerve innervation varies among different tissue types and species, additional research in diverse tissues and larger animal models is necessary to corroborate our findings.

In conclusion, this study demonstrates that local adrenergic β-receptor blockade, rather than systemic administration, can effectively enhance burn wound treatment. We anticipate that scaffolds with agents inhibiting sympathetic activation will hold significant promise for tissue engineering applications.

## 5 Conclusion

In summary, we have successfully developed a novel anti-sympathetic PNL@GA-Cu hydrogel to address the sustained stress induced by burns. This hydrogel boasts multifunctional properties, including injectability and antibacterial activity. The gradual release of copper ions throughout the healing process effectively eliminates infections caused by *E. coli* and *S. aureus*. When subjected to NE-mimicking sympathetic nervous system activation, the PNL@GA-Cu hydrogel significantly enhances HUVEC proliferation, migration, and angiogenesis. Furthermore, the sustained release of propranolol in the PNL@GA-Cu hydrogel inhibits PC12 cell migration and tyrosine hydroxylase expression, while also counteracting NE binding to target cells. Additional in vivo studies confirm that the PNL@GA-Cu hydrogel promotes the formation of new blood vessels in treated wounds while delaying the innervation of new sympathetic nerves. Importantly, compared to the Control, GA-Ca, and GA-Cu groups, the newly formed skin treated with PNL@GA-Cu hydrogel exhibits a greater number of skin appendages, rendering it a promising therapeutic approach for healing infected burned wounds.

## CRediT authorship contribution statement

**Wenzhe Sun:** Writing – original draft, Investigation, Formal analysis. **Hongwei Lu:** Methodology, Formal analysis. **Pengqin Zhang:** Investigation. **Lian Zeng:** Investigation. **Bing Ye:** Project administration. **Yi Xu:** Conceptualization. **Jianan Chen:** Conceptualization. **Peiran Xue:** Conceptualization. **Jialin Yu:** Conceptualization. **Kaifang Chen:** Supervision, Funding acquisition. **Bin Wu:** Funding acquisition, Conceptualization. **Xiao Lv:** Supervision, Funding acquisition, Conceptualization. **Xiaodong Guo:** Supervision, Funding acquisition, Conceptualization. **Yanzhen Qu:** Supervision, Funding acquisition, Conceptualization.

## Permission to reproduce material from other sources

Figures were all created with BioRender.com, and publication licenses were obtained.

## Declaration of generative AI and AI-assisted technologies in the writing process

AI was not used during the preparation of this work.

## Funding sources

This work was supported by the 10.13039/501100001809National Natural Science Foundation of China (Grant No. 82102546, 82102627, 82202715, 82072446, 82272460) and the 10.13039/501100003819Natural Science Foundation of Hubei Province (2021CFB277, 2021CFB596).

## Declaration of competing interest

The authors declare the following financial interests/personal relationships which may be considered as potential competing interests:Yanzhen Qu reports financial support was provided by 10.13039/501100001809National Natural Science Foundation of China. Xiaodong Guo reports financial support was provided by 10.13039/501100001809National Natural Science Foundation of China. Xiao Lv reports financial support was provided by 10.13039/501100001809National Natural Science Foundation of China. Kaifang Chen reports financial support was provided by 10.13039/501100001809National Natural Science Foundation of China. Bin Wu reports financial support was provided by 10.13039/501100003819Natural Science Foundation of Hubei Province. Yanzhe Qu reports financial support was provided by 10.13039/501100003819Natural Science Foundation of Hubei Province. If there are other authors, they declare that they have no known competing financial interests or personal relationships that could have appeared to influence the work reported in this paper.

## Data Availability

Data will be made available on request.

## Abbreviations

PNL: Propranolol
β-ARs: β-adrenergic receptors
NE: Norepinephrine
HUVECs: Human vascular endothelial cells
TH: Tyrosine hydroxylase
EPA: Environmental Protection Agency
ROS: Reactive oxygen species
QS: Quorum Sensing
Sema3A: Semaphorin 3A
