# Supplementary material for: Localized propranolol delivery from a copper-loaded hydrogel for enhancing infected burn wound healing via adrenergic β-receptor blockade
Source: Mater Today Bio. 2024 Dec 20;30:101417. doi: 10.1016/j.mtbio.2024.101417 (PMC11785566; doi:10.1016/j.mtbio.2024.101417)
Supplement: Multimedia component 1 [file mmc1.docx]

**Figure S1.** Cell viability of HUVECs cultured with PNL@GA-Cu extracts for 24 hours, where the hydrogel was cross-linked with 1.0%, 1.5%, 2.0%, and 2.5% (w/v) CuSO_4_ solution for 5 minutes.


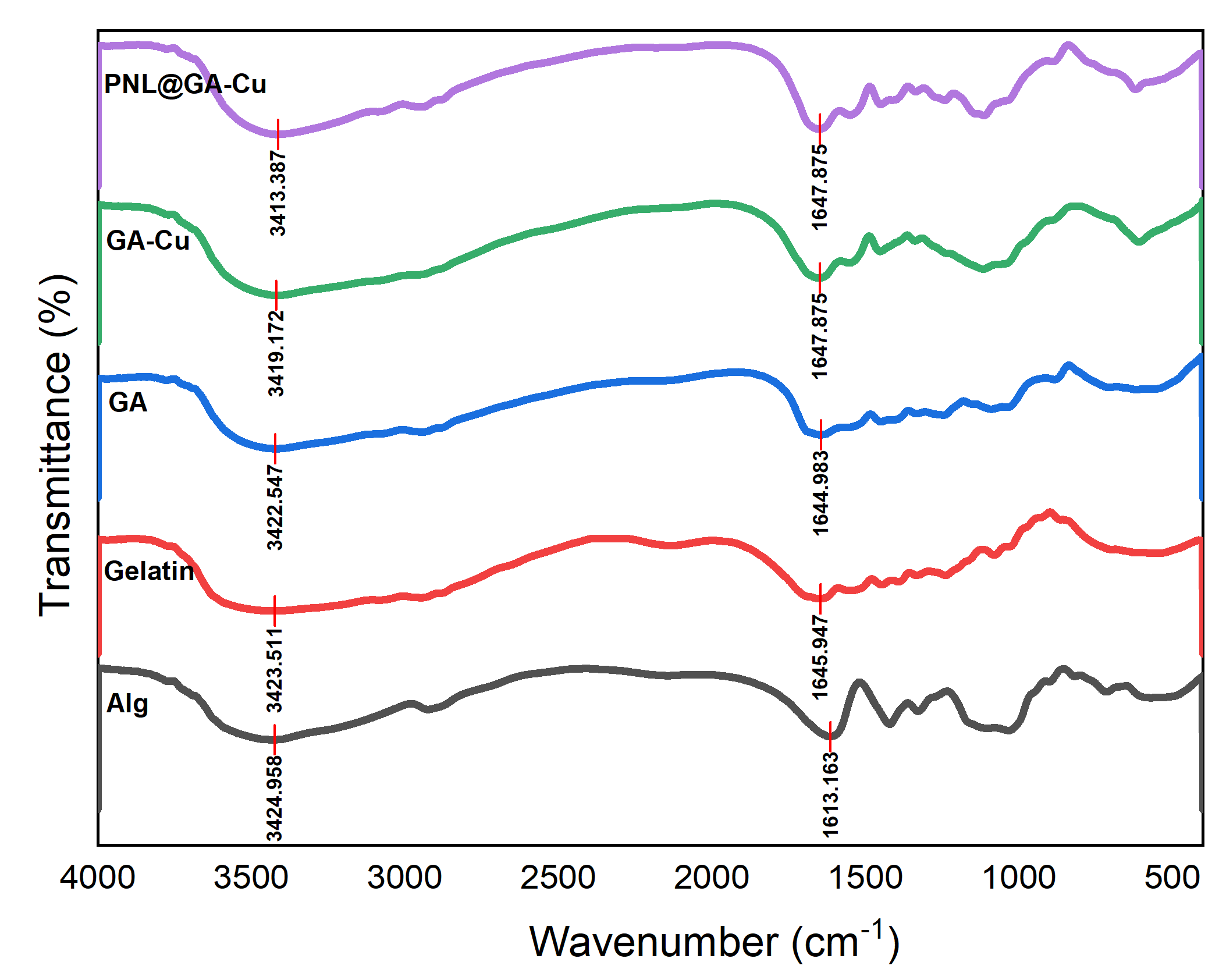


**Figure S2.** FTIR spectra of GA, GA-Cu, and PNL@GA-Cu hydrogels.


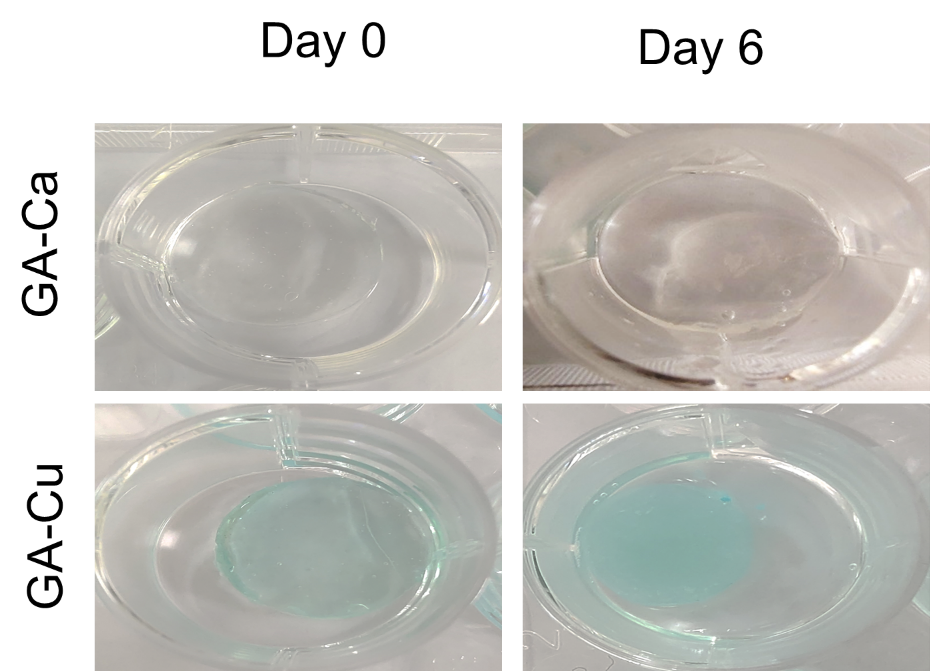


**Figure S3.** Degradation of GA-Ca and PNL@GA-Cu hydrogels in PBS at 37℃.


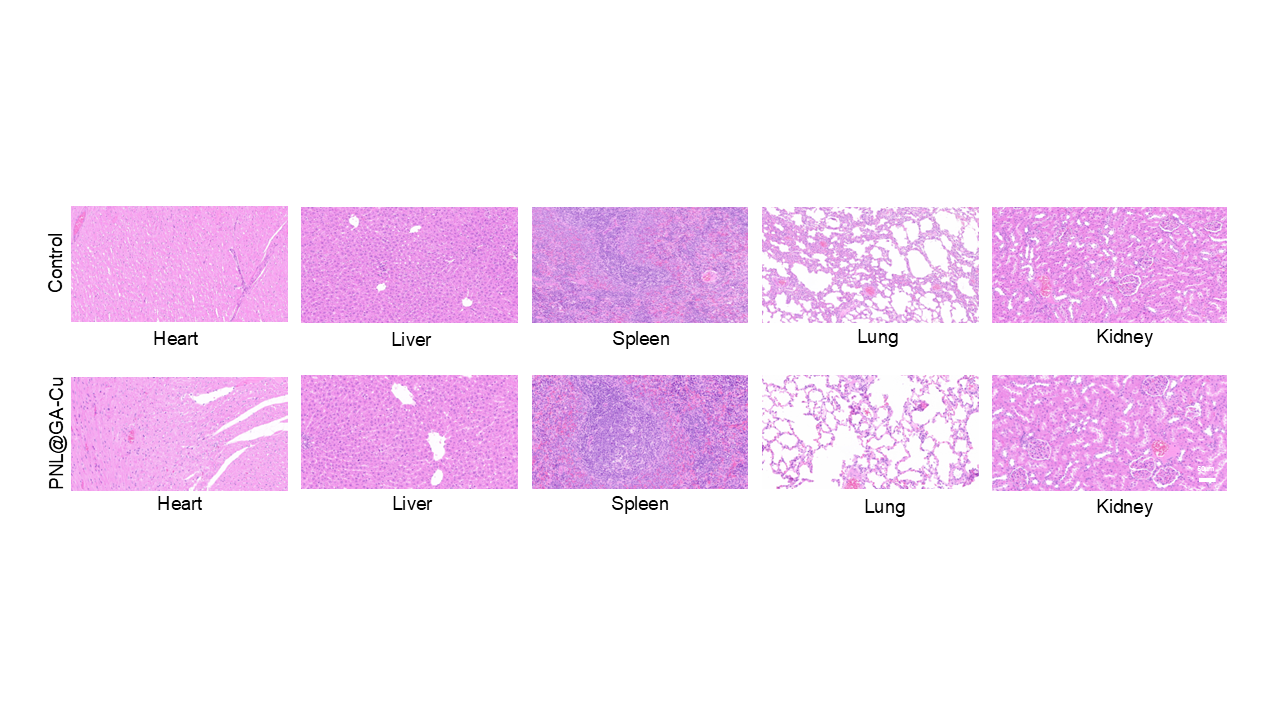


**Figure S4.** H&E staining analysis of heart, liver, spleen, lung, and kidney from the rats treated with PBS or PNL@GA-Cu hydrogel.


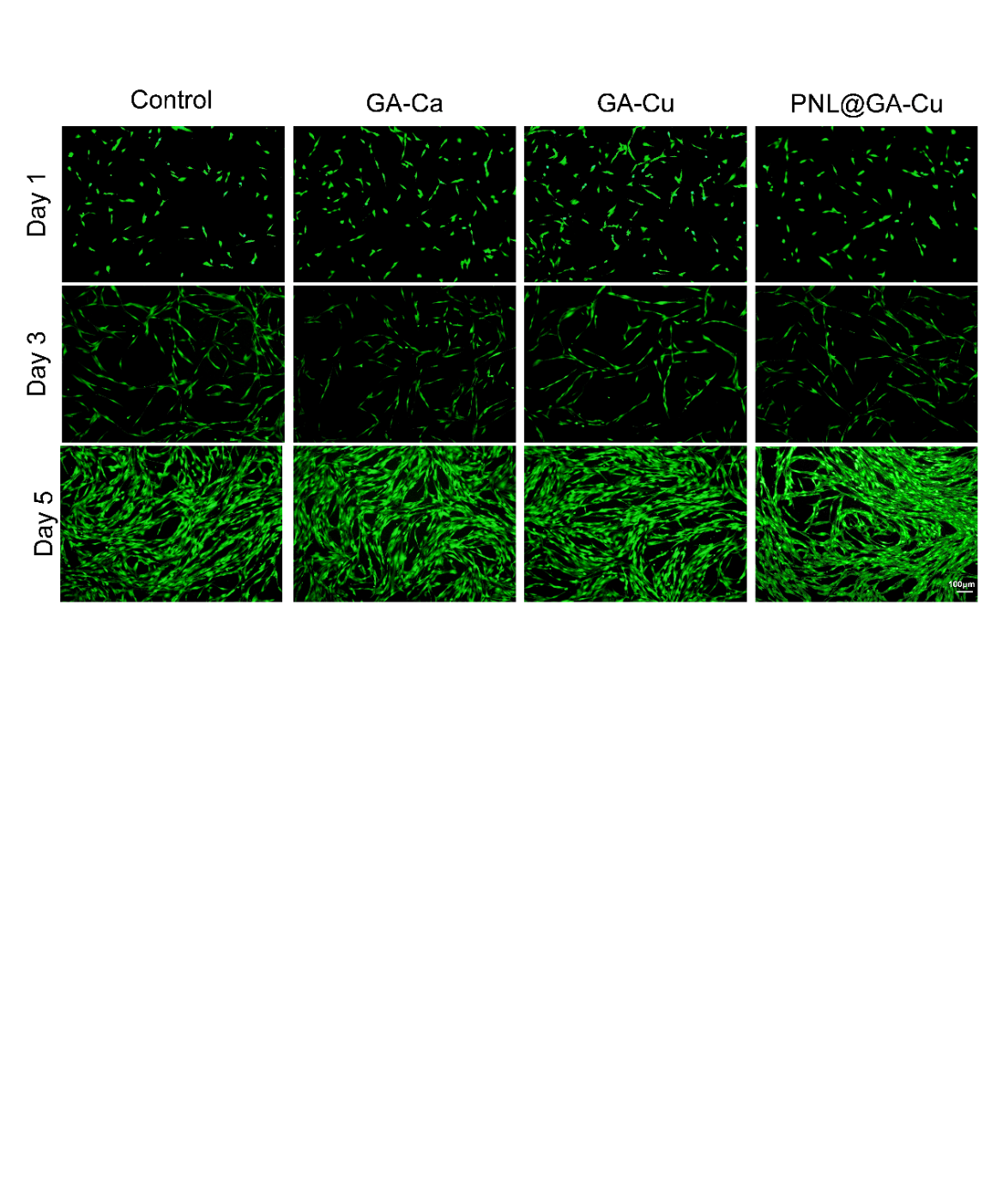


**Figure S5.** Live/dead staining results for different groups of PC12 cells over 1, 3, and 5 days.

**Figure S6.** Viability of PC12 cells treated with the different types of hydrogels on days 1, 3, and 5.


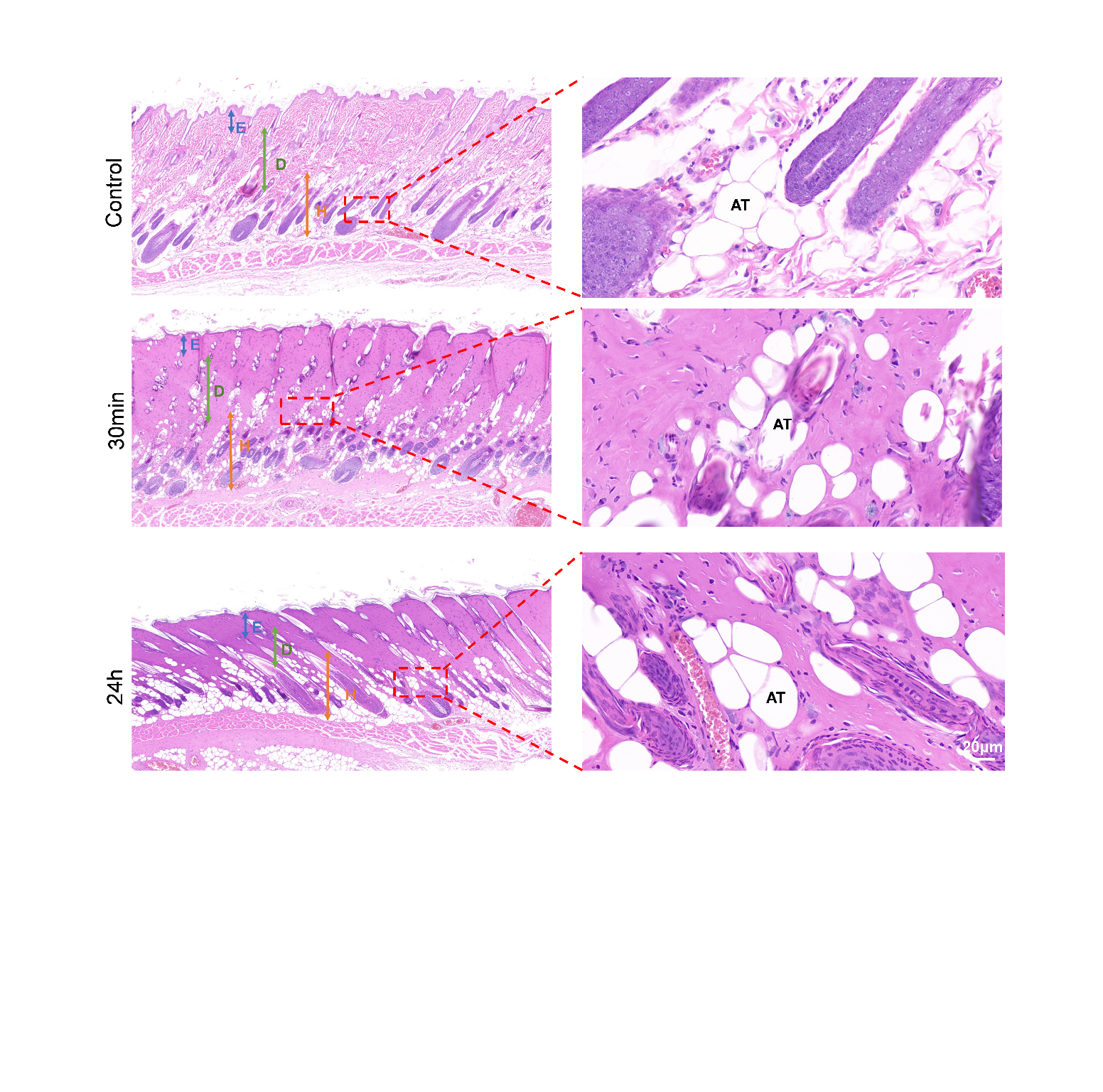


**Figure S7.** Representative images of normal skin and wounds on day 1 stained with H&E; E, epidermis; D, dermis; H, hypodermis; AT, adipose tissue.
